# A Comprehensive In Silico Exploration of Pharmacological Properties, Bioactivities, Molecular Docking, and Anticancer Potential of Vieloplain F from *Xylopia vielana* Targeting B-Raf Kinase

**DOI:** 10.3390/molecules27030917

**Published:** 2022-01-28

**Authors:** Syed Shams ul Hassan, Syed Qamar Abbas, Fawad Ali, Muhammad Ishaq, Iqra Bano, Mubashir Hassan, Hui-Zi Jin, Simona G. Bungau

**Affiliations:** 1 Shanghai Key Laboratory for Molecular Engineering of Chiral Drugs, School of Pharmacy, Shanghai Jiao Tong University, Shanghai 200240, China; shams1327@yahoo.com (S.S.u.H.); mishaqjnj@yahoo.com (M.I.); 2 Department of Natural Product Chemistry, School of Pharmacy, Shanghai Jiao Tong University, Shanghai 200240, China; 3 Department of Pharmacy, Sarhad University of Science and Technology, Peshawar 25000, Pakistan; qamar0613@yahoo.com; 4 Department of Pharmacy, Kohat University of Science and Technology, Kohat 26000, Pakistan; fawadalee@gmail.com; 5 Faculty of Bio-Sciences, SBBUVAS, Sakrand 67210, Pakistan; iqrashafi05@yahoo.com; 6 Institute of Molecular Biology and Biotechnology, The University of Lahore, Nisbet Road, Lahore 54000, Pakistan; mubashirhassan_gcul@yahoo.com; 7 Battelle Center for Mathematical Medicine, The Research Institute at Nationwide Children’s Hospital, Columbus, OH 43205, USA; 8 Department of Pharmacy, Faculty of Medicine and Pharmacy, University of Oradea, 410028 Oradea, Romania; simonabungau@gmail.com

**Keywords:** guaiane dimer, melanoma, molecular docking, ADMET, MM-GBSA

## Abstract

Compounds derived from plants have several anticancer properties. In the current study, one guaiane-type sesquiterpene dimer, vieloplain F, isolated from *Xylopia vielana* species, was tested against B-Raf kinase protein (PDB: 3OG7), a potent target for melanoma. A comprehensive in silico analysis was conducted in this research to understand the pharmacological properties of a compound encompassing absorption, distribution, metabolism, excretion, and toxicity (ADMET), bioactivity score predictions, and molecular docking. During ADMET estimations, the FDA-approved medicine vemurafenib was hepatotoxic, cytochrome-inhibiting, and non-cardiotoxic compared to the vieloplain F. The bioactivity scores of vieloplain F were active for nuclear receptor ligand and enzyme inhibitor. During molecular docking experiments, the compound vieloplain F has displayed a higher binding potential with −11.8 kcal/mol energy than control vemurafenib −10.2 kcal/mol. It was shown that intermolecular interaction with the B-Raf complex and the enzyme’s active gorge through hydrogen bonding and hydrophobic contacts was very accurate for the compound vieloplain F, which was then examined for MD simulations. In addition, simulations using MM-GBSA showed that vieloplain F had the greatest propensity to bind to active site residues. The vieloplain F has predominantly represented a more robust profile compared to control vemurafenib, and these results opened the road for vieloplain F for its utilization as a plausible anti-melanoma agent and anticancer drug in the next era.

## 1. Introduction

Melanoma is the most aggressive and deadly form of skin cancer [1,2,3,4]. It is the seventh leading cancer in women and fifth in men in the USA [5,6]. It is predicted that the five-year survival rate for patients with stage IV or advanced melanomas is less than 15%. This cancer can grow and spread for an indefinite period due to mutations in the cells [7]. The primary treatments are BRAF, C-Kit, and NRAS inhibitors. BRAF inhibitors, such as vemurafenib, are the most effective FDA-approved treatments for BRAF positive melanoma. However, the negative side of the drug vemurafenib is that patients started getting resistance after six months of therapy, making therapy no longer effective [8]. More effective treatments are urgently needed as the prevalence of melanoma rises in the United States and other developed nations.

There has been an increase in scientific interest in medicinal plants [9], In the last several decades, plants with their intriguing secondary metabolites have been explored for their strong anticancer properties [10,11]. The family Annonaceae includes the genus Xylopis. Recently, it has been described for its extraordinary broad range of pharmacological spectrum encompassing rheumatism, analgesia, bactericidal, fungicidal, antioxidant antitumor and anti-inflammatory properties. *Xylopia vielana* is the only species identified in China [12,13,14,15,16]. Natural products have been shown to influence BRAF kinases; however, it is unclear whether or not they communicate with the gene product or even the transposable elements that regulate the gene. [5]. 

The manufacturing of a new medicine is a lengthy and complex procedure. Selecting a suitable lead molecule is one of the crucial steps during drug development [17]. However, unexpected toxicity and adverse drug reactions, on the other hand, caused around 40% of the drug candidates to fail [18]. Computer-assisted in silico approaches have become increasingly important in the initial phases of drug development as they are more cost-effectively [17,19]. This method decreases the number of animals killed and reduce failures in the ultimate stage [19]. Often, unexpected toxicity is detected late in drug development. An in silico technique for predicting toxicological parameters is an alternative to animal testing [20]. The guaiane dimers from *Xylopia vielana* due to their complex unique structures are already proved to provide great effects during in silico studies against CoX-2 [12]. During the in silico studies such as ADMET, the initial profile of the compounds has been created. If any of the compounds provide any severe toxicity or create drug-drug interaction with some metabolizing cytochromes, the time should not be wasted on that kind of compounds. 

New dimers were discovered in our recent study on the *Xylopia vielana* to aid in the quest for powerful anticancer medicines [13,15]. The in vitro anticancer potential of these guaiane dimers has been reported, which shows that some of these guaiane dimers, especially vieloplain F have displayed potent anticancer activity with IC_50_ values of 9.5 µM. However, its profound mechanistic studies and pharmacological profile were incomplete. Vieloplain F’s pharmacological characteristics were studied in this study, with an emphasis on drug-likeness, bioactivities, administration, distribution, metabolic activity, excretion, and toxicity. The guaiane dimer blockage of BRAF kinases was further studied using molecular docking, MD modeling, and MM-GBSA calculations to identify its carcinogenic mechanism.

## 2. Results

### 2.1. Chemical Structures of Vieloplain F and Vemurafenib 

**Figure 1:**
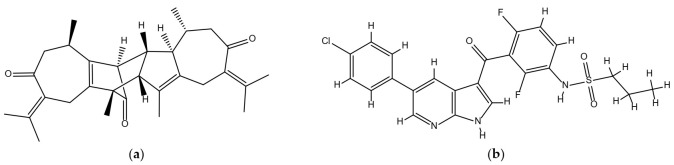
Chemical structures of (**a**) vieloplain F; (**b**) vemurafenib.

The chemical structure of a guaiane dimer vieloplain F was drawn on ChemBioDraw (v13.0) and the control drug vemurafenib was downloaded from PubChem and was redrawn on ChemBioDraw (Figure 1).

### 2.2. Estimation of Activity Spectra for Substances (PASS) 

Over 4000 biological activities are predicted by PASS Online (Way2Drug, Moscow, Russia), including pharmacological effects, modes of action, toxic and unfavorable consequences, linkages with metabolic enzymes and transporter, and impact on expression of genes. Table 1 shows the best outcomes from all of the predicted activities for vieloplain F. The biological activity as antineoplastic had the most significant predicted activity (Pa) for vieloplain F, with Pa > 0.8. The probable activity (Pa) values were higher than Pa > 0.5, and the probable inactivity (Pi) scores were extremely near to 0, demonstrating that the compound is highly expected to demonstrate these activities.

### 2.3. Toxicological and Pharmacokinetic Assets

Absorption, distribution, metabolism, excretion, and toxicity (ADMET) estimations were primarily directed at all the guaiane dimers (vieloplains A–G). Many novel compounds have been exemplified during initially ADMET screening due to violations, including inhibiting different cytochromes, skin permeation, and P-gp inhibition. Only vieloplain F was selected for further studies because of no violations. 

#### 2.3.1. Pharmacokinetic Characteristics 

**Figure 2:**
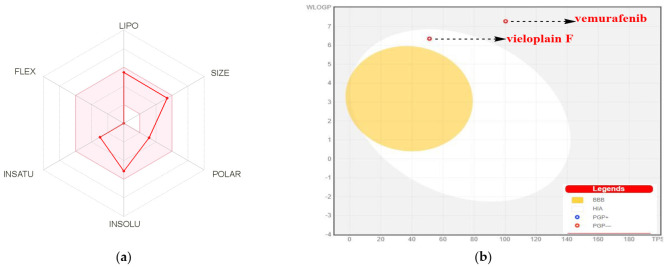
(**a**) Bioavailability radar chart for vieloplain F. The pink zone represents the physicochemical space for oral bioavailability, and the red line represents the oral bioavailability properties. (**b**) Predicted BOILED-Egg plot from *swiss ADME* online web tool for all the compounds.

The physico-chemical characteristics of both compounds are discussed in Table 2. According to Table 2, the lipophilicity, insolubility, size, insaturation, polarity, and flexibility of vemurafenib and vieloplain F were studied and classified into six sections with appropriate ranges for oral bioavailability (Figure 2a). The oral bioavailability graph of the vieloplain F is shown in Figure 2a and vemurafenib in Figure S1 (Supplementary Materials), which is based on the six sections stated in the physicochemical characteristics section. The results of the compound vieloplain F was within these limits, demonstrating that vieloplain F has a favorable physiochemical profile, which is one of the factors that must be monitored in pharmaceuticals and clinical studies. 

HIA and CNS absorption are important parameters checked for every biomolecule before its entry for drug formulation in the pharmaceutical or clinical trials field [21]. The blood–brain barrier penetration is essential as if the compounds that act on the central nervous system (CNS) must cross through the blood–brain barrier, and the inactive compounds on the CNS should not intersect to avoid adverse effects on the CNS [22]. As mentioned in Table 3, the compound vieloplain F displayed a high gastrointestinal absorption (HIA) with no BBB permeability, indicating that vieloplain F shows low occurrence for adverse CNS effects. The compound vieloplain F HIA absorption ratio was elevated than vemurafenib. 

Figure 2b shows the BOILED-EGG curve [23]. The BBB penetration and GI absorption (HIA) of the substances may be predicted by this method. There are two areas: one for the GI absorption zone (HIA) and the other for BBB penetration (yolk). Neither GI absorption nor BBB penetration is indicated if any component is found in the gray zone. Because neither vieloplain F nor the control medication vemurafenib showed that they are P-gp substrates, they are not sensitive to the efflux mechanism of P-gp, which is used by many cancers’ cell lines to develop resistance to drugs. Vemurafenib, the reference medicine, was shown in gray, whereas vieloplain F was shown in white, as can be seen in Figure 2b.

The skin permeation Log K_p_ of vieloplain F, compared to vemurafenib, was lower (Table 3), as mentioned by [24]. The greater the negative value of K_p_, the less permeant the molecule is to the skin. It also forecasts the five main cytochromes (CYP) isoform, which is an additional benefit. These enzymatic isoforms play a crucial role in the excretion of pharmaceuticals, and they handle the metabolism of about 75% of the medicines available on the market. There are major drug-drug interactions caused by inhibiting any of these isoforms [21,24]. Compared to the control vemurafenib, the vieloplain F did not block any cytochrome isoform, as shown in Table 3, and was rapidly metabolized. Drug-medication interactions may occur when three cytochrome isoforms are inhibited by the control drug vemurafenib: CYP2C19, CYP2C9, and CYP3A4. Dosing rates for achieving steady-state concentrations depend on drug clearance, which is determined by adding up the excretion rates from the liver and kidney. Moreover, vieloplain F’s clearance value was insufficient. Organic cation transporter 2 (OCT2) intermediates may have an influence on the unfavorable interactions that occur when OCT2 inhibitors and substrates are used together. It has been hypothesized that the compound vieloplain F would act as a non-substrate for OCT2.

#### 2.3.2. Toxicity Assessment

It is critical to evaluate the toxicological profile of a medicine before it reaches the clinical trials stage or the production phase of the pharmaceutical business before it is approved [25]. A variety of toxicities were assessed for each molecule, including those affecting human health and those affecting the environment. (Table 4). Using the Ames test, a compound’s mutagenic potential may be assessed. Both substances were classified as non-Ames dangerous, meaning that they are uncertain to be carcinogenic, according to the results. In humans, the maximum tolerated dose (MTD) serves as an indicator of a chemical’s toxicity level. In comparison to vieloplain F, the MTD for vemurafenib was significantly greater. It is possible that inhibition of the potassium channels encoded by the hERG would cause a catastrophic ventricular arrhythmia. Several studies have shown that both vieloplain F and vemurafenib can inhibit hERG II, but not hERG I. However, vemurafenib was expected to be hepatotoxic, which would likely result in drug-induced liver damage. Vieloplain F was also predicted to be non-hepatotoxic. Skin hypersensitivity is a possible adverse effect of dermally given products, and none of the chemicals tested has been shown to cause skin sensitization in humans.

In addition, for the prediction of lethal dose (LD_50_), the compound vieloplain F received a score greater than 300 mg/kg and was classified as class 4; therefore, it is considered “harmful if swallowed” (300 < LD_50_ ≤ 2000), while the control vemurafenib has a score of over 2000, and classified as class 5; therefore, it is considered “may be harmful if swallowed” (2000 < LD_50_ ≤ 5000), see Table 4. Vieloplain F and vemurafenib, according to their toxicological qualities, are not considered being at risk for protein toxicity, and the substances are classified as classes 4 and 5.

GUSAR, an online web server (Way2Drug, Moscow, Russia), and the environmental toxicity of each molecule were all considered. GUSAR predicted the environmental toxicity, where 96-h fathead minnow 50% lethal concentration, 48-h *Daphnia magna* 50% lethal concentration, and *Tetrahymena pyriformis* 50% growth inhibition concentration and bioconcentration factors were evaluated. The results are depicted in Table 4. The chemicals vieloplain F and vemurafenib fit into the application area of models in all circumstances when it comes to environmental toxicity prediction using GUSAR. Because of its superior safety profile when compared to vemurafenib, guaiane dimer vieloplain F was shown to have a lower risk of liver damage.

### 2.4. Drug-Likeness Prediction 

The drug-likeness explains the compound’s potential as a drug molecule candidate. As shown in Table 2 and Table S1, the compound vieloplain F met the requirements of drug-likeness and passed their filters, such as the Veber filter (rotatable bonds ≤ 10 with TPSA ≤ 140) [26]. Furthermore, the compound vieloplain F was also checked for Lipinski’s rule of five (MW ≤ 500, MLOGP ≤ 4.15, N or O ≤ 10, NH or OH ≤ 5 and Log *P_o/w_* ≤ 5), except for one violation of Lipinski’s rule of five with Log *P_o/w_* greater than 5 (Table 2) [27]. In addition, both vieloplain F and vemurafenib displayed a good bioavailability score of 0.55, within the range of F > 10% in rats which further proves the Veber and Lipinski’s rule of five predictions [28]. Importantly, neither vieloplain F nor vemurafenib elicited an indication for the pan assay interference substances (PAINS), demonstrating that neither medication contains any fragments that might cause false positive biological tests. The molecules that must be synthesized in the laboratory are critical if they are to be produced in large quantities. The structure’s intensity is graded into three categories: moderate (scores 1–4), medium (scores 4–7), and difficult (scores 8–10) [24]. The combination vieloplain F has received a score of 6.46 (Table S1, Supplementary Materials), which shows that vieloplain F, according to its complicated structure, is difficult to synthesize in the laboratory, but vemurafenib is easier to produce. In conclusion, these in silico studies for modeling the physicochemical and pharmacokinetic properties of vieloplain F revealed that the pattern of vieloplain F was better than that of vemurafenib in terms of CYP inhibition, hepatotoxicity, and pharmacokinetic properties. Vieloplain F was also found to have superior pharmacokinetic properties. 

### 2.5. Prediction of the Bioactivity Score

Using the Molinspiration Chemoinformatics tools, the projected bioactivity scores of the substances vieloplain F and vemurafenib were calculated. These results are shown in Table 5. According to the bioactivity score, vieloplain F is moderately active in the presence of a G-protein-coupled receptor (GPCR) ligand, an intracellular signaling regulator, a kinase inhibitor, as well as protease inhibitors, while vemurafenib is moderately active. According to studies by [29] the nuclear receptor has a dual function in inflammation and immunity. Vieloplain F is a more dynamic and high-scoring molecule than the control vemurafenib, according to the research. Enzyme inhibition levels show both substances were active. Vieloplain F’s activity score profile shows it is physiologically active and has a physiologic impact. There has been no inactivity, as predicted by the bioactivity score. 

### 2.6. Prediction of Cardiac Toxicity

**Figure 3:**
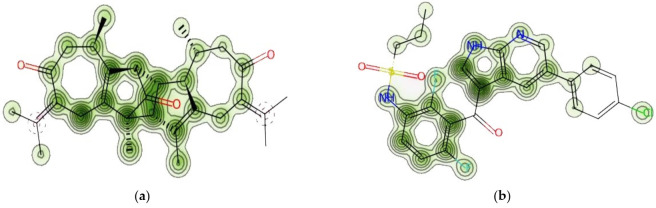
Cardiac toxicity of drugs derived from pred-hERG in a map format: (**a**) vieloplain F; (**b**) vemurafenib.

The FDA requires that every biomolecule be tested for hERG safety before it may be used as a therapeutic candidate. The hERG blockage has been connected to deadly cardiac arrhythmias. Using pred-hERG results to predict cardiac toxicity, the likelihood map for vieloplain F and vemurafenib as a control is shown here (Figure 3). Attributions to hERG blockage, both positive and negative, are shown in the figure. Increasing the number of contour lines and the intensity of the green color shows that an atom or fragment has made a more positive contribution to the hERG blockage. With a 50% confidence level, the pred-hERG projected that Vieloplain F would be non-cardiotoxic, whereas the control vemurafenib was projected as having a 60% confidence level that it may be cardiotoxic. The findings have revealed that our isolated molecule, vieloplain F, is less hazardous to the heart than the control drug, vemurafenib, for cardiovascular toxicity.

### 2.7. Biomolecular Macromolecules: Epoxidation and Reactivity Prediction

**Figure 4:**
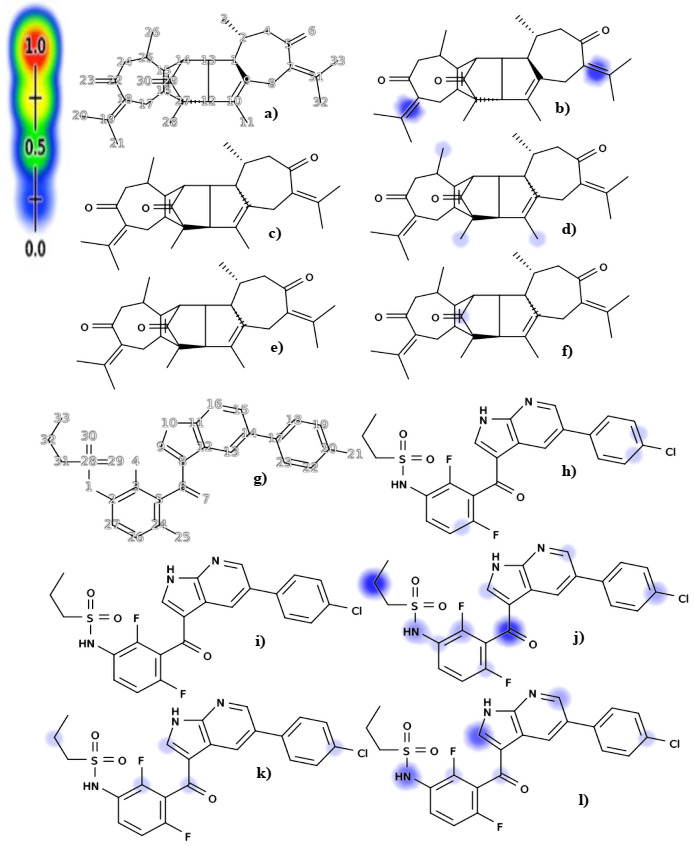
Prediction of epoxidation and reactivity to biological macromolecules by XenoSite. (**a**) The input structure of vieloplain F; (**b**) prediction of epoxidation; prediction of reactivity to: (**c**) cyanide; (**d**) DNA; (**e**) glutathione (GSH); (**f**) protein; (**g**) the input structure of vemurafenib; (**h**) prediction of epoxidation; prediction of reactivity to: (**i**) cyanide; (**j**) DNA; (**k**) glutathione (GSH); (**l**) protein.

The prediction of epoxidation and reactivity to biological macromolecules of vieloplain F and control vemurafenib is depicted in Figure 4. The possible sites of epoxidation of the compound vieloplain F have been shown in Figure 4b. Here, the double bond between atoms 18–19 and 31–7 was more prone to epoxidation, where the probability score was 0.19. In Figure 4c,e, cyanide and GSH’s reactivity showed no probability scores for any atoms. In Figure 4d, reactivity to DNA is shown for atoms 11, 26, and 28 with the probability scores of 0.050, 0.029, and 0.030. In Figure 4f, reactivity to protein was only shown for one atom 29 with a probability score of 0.047. The possible sites of epoxidation of the control vemurafenib are shown in Figure 4h. Here, the epoxidation was predicted on one double bond between atoms 19–20 with a probability score of 0.031. The epoxidation was also predicted on atoms between 20–22 and 24–26 with the probability score of 0.031 and 0.035. In Figure 4i, cyanide reactivity showed no probability scores for any atoms. In Figure 4j, reactivity to DNA is shown for nine atoms, among which the highest probability score was predicted for atoms 6 and 32 with the score of 0.21. The reactivity to DNA for the rest atoms 1–3, 9, 15, 20, and 24 shown probability scores with 0.11, 0.066, 0.081, 0.038, 0.029, 0.075, and 0.028. In Figure 4k, reactivity to GSH was shown for atoms 3, 6, 9, 20, and 32 with the probability score of 0.031, 0.056, 0.053, 0.050, and 0.045. In Figure 4l, reactivity to protein was shown for the atoms 1, 3, 6, 9, 15, and 20 with probability scores of 0.14, 0.057, 0.050, 0.17, 0.078, and 0.034, in which the highest score was predicted for atom 9. The results declared that the guaiane dimer vieloplain F has better reactivity results than control vemurafenib, which also has better reactivity results, and prove that natural compounds have better epoxidation and reactivity profiles.

### 2.8. Prediction of Endocrine Disruption Potential

**Figure 5:**
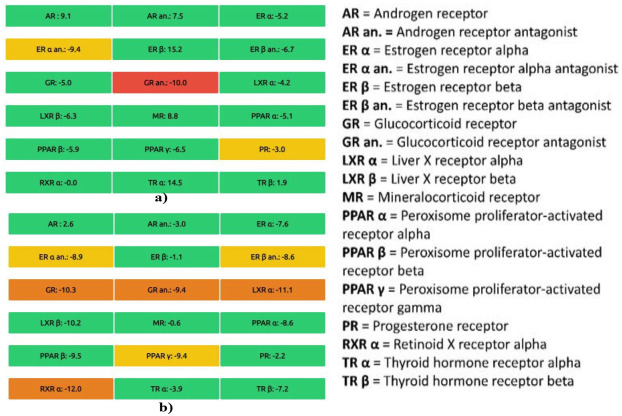
Endocrine disruption potential of compounds as obtained from Endocrine Disruptome. (**a**) Vieloplain F, (**b**) vemurafenib. Red color describes the high probability of binding, Orange and Yellow describes the medium probability of binding while the Green color describes the low probability binding.

The multi-color-coded table obtained from the online web tool of Endocrine Disruptome is given in Figure 5. There were fourteen nuclear receptors with eighteen targets. For the compound vieloplain F, according to the results (Figure 5a), fifteen targets showed low probability as they were coded in green (sensitivity > 0.75). Two targets, estrogen receptor alpha antagonist and progesterone receptor, were coded in yellow, indicating that vieloplain F had a medium probability of binding (0.50 < sensitivity < 0.75), and one target, glucocorticoid receptor antagonist, was coded in red, indicating a high probability of binding (sensitivity < 0.25). The control vemurafenib showed the most negligible results compared to vieloplain F Figure 5b. Initially, only 11 targets were coded green (sensitivity > 0.75) and showed a low probability. Three targets were encompassed in the yellow zone, such as estrogen receptor alpha antagonist, estrogen receptor beta antagonist, and peroxisome proliferator-activated receptor gamma, indicating that vemurafenib had a medium probability of binding (0.50 < sensitivity < 0.75). Four targets were encompassed in orange color (0.25 < sensitivity < 0.50)—glucocorticoid receptor, glucocorticoid receptor antagonist, liver X receptor alpha, and retinoid X receptor alpha—indicating a medium probability of binding. The control did not show any high probability of binding to the nuclear receptor. The results have revealed that vieloplain F has a strong profile as it has only one target encoded in red color, but still, the docking score of that target is −10.0, which is a high score for binding. According to docking rules, more negative scores mean more affinity towards binding. As mentioned in Figure 5b, the control vemurafenib has very high orange zone scores up to −12.0, indicating a higher possibility for bindings. From all the results, it has been clear that the control vemurafenib has shown more human nuclear receptors binding affinity than vieloplain F.

### 2.9. Prediction of Cell Line Cytotoxicity

In silico prediction of cell line cytotoxicity for cancer cells is shown in Table 6. Both the compounds vieloplain F and control vemurafenib showed the highest scores for the melanoma cell line (Sk-Mel-28). In this set of predictions, probable activity (Pa) was higher than probable inactivity (Pi), and we only selected Pa > 0.5 because the differences were significant; these results can be recorded as probable cytotoxic activities for both the compounds. Vieloplain F was recorded with the highest score of (Pa) 0.722 against melanoma.

### 2.10. Docking of the Compounds with B-Raf Kinase Structure (PDB: 3OG7)

#### 2.10.1. Structural Analysis of B-Raf Kinase

B-Raf kinase is a class of transferase that consists of two domains having 289 amino acids. The overall statistical VADAR analysis showed protein architecture, containing 39% helices, 22% β sheets, and 38% coils. Moreover, Ramachandran plots indicated that 99.6% of residues were present in the allowed region, which shows the precision of phi (φ) and psi (ψ) angles among the coordinates of B-Raf kinase (Figure S2, Supplementary Materials).

#### 2.10.2. Binding Energy Evaluation of the Compounds 

To predict the best conformational position within the active region of the target protein, vieloplain F and vemurafenib were docked and analyzed based on docking energy value (kcal/mol). Moreover, generated docked complexes were examined based on the hydrogen/hydrophobic interaction pattern. Docking results justified that the compound vieloplain F showed good energy values (−11.8 kcal/mol) as compared to standard vemurafenib (−10.2 kcal/mol) against target protein (Table 7). The comparative analysis showed that guaiane dimer vieloplain F may have good therapeutic potential against B-Raf kinase protein and can be considered an emerging candidate against melanoma.

#### 2.10.3. Protein–Ligand Complex Analysis 

**Figure 6:**
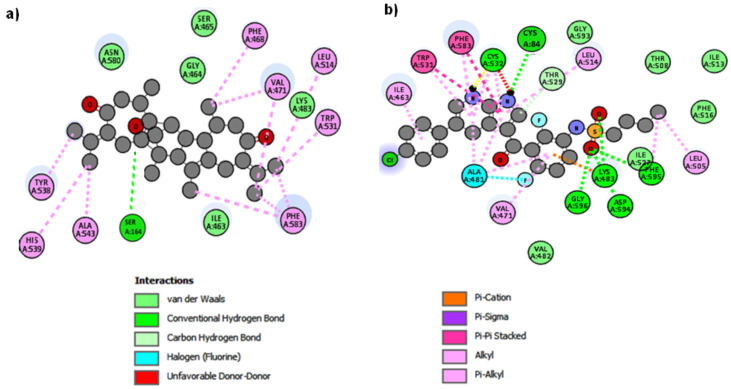
(**a**) 2D interactions of the compound vieloplain F with B-Raf (PDB: 3OG7); (**b**) 2D interactions of the control vemurafenib with B-Raf (PDB: 3OG7).

The compounds vieloplain F and vemurafenib were bound against the target protein in different conformations. In vieloplain F docking results, a single hydrogen bond was observed at SER164. The benzene oxygen atom forms a hydrogen bond, with SER164 having a bond length of 2.29 Å. The comparative results showed that common residues were observed in docking studies, strengthening our docking results (Figure 6a). In vemurafenib docking results, hydrogen bonds were observed at CYS84, CYS532, LYS483, GLY596, ASP594, and PHE595. The residual comparison showed that our compound binds within the target protein’s active site as with standard drug vemurafenib with different conformations (Figure 6b).

### 2.11. Molecular Dynamics and Simulation

MDS was performed for the vemurafenib-B-Raf kinase structure complex and vieloplain-F- B-Raf kinase structure complex up to 100 ns. The parameters explored for analyses include RMSD, RMSF, protein–ligand contact, RoG, and binding free energy.

#### 2.11.1. RMSD Analysis

**Figure 7:**
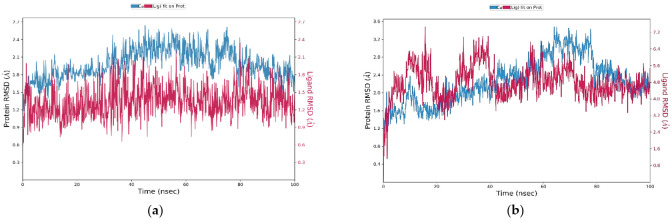
Root mean square deviation (RMSD) of the (**a**) vemurafenib–B-Raf kinase; (**b**) vieloplain-F–B-Raf kinase.

The RMSD study was done to find the simulation results stabilities. The RMSD graph of protein (left Y-axis) can give the understanding of its structural conformation during the simulation while ligand RMSD (right Y-axis) denotes the stability of ligand toward the specific protein and its binding site pocket. In the case of vemurafenib-B-Raf kinase complex, initially, RMSD showed robust stabilization and remained stable throughout the simulation period up to 100 ns. The vieloplain-F–B-Raf kinase complex showed that both vieloplain-F and B-Raf Kinase attained stability at 20 ns and remained constant throughout the simulation (Figure 7A). At first, the vieloplain-F–B-Raf kinase complex RMSD showed fluctuations of 0.2 nm up to 20 ns and then remained stable through the simulation period up to 100 ns (Figure 7B). Both complexes revealed a good level of interaction throughout the simulation, with less deviation in structure.

#### 2.11.2. RMSF Assay

**Figure 8:**
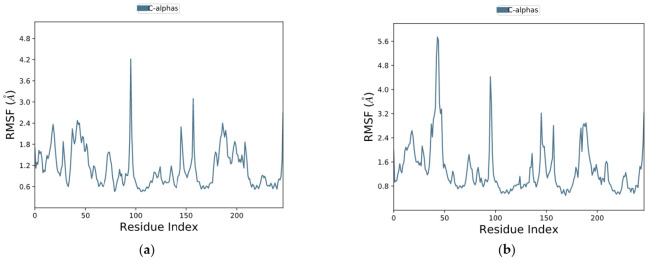
Root mean square fluctuation (RMSF) of protein complexes (**a**) vemurafenib–B-Raf kinase, (**b**) vieloplain-F–B-Raf kinase.

**Figure 9:**
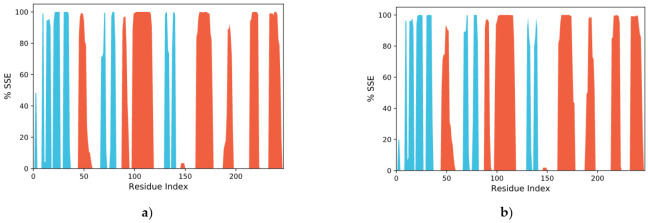
Protein secondary structure element distribution by residue index throughout the protein structures complexed with ligand (**a**) vemurafenib–B-Raf kinase, (**b**) vieloplain-F–B-Raf kinase. The red color represents α-helices, and the blue represents β-strands.

The RMSF defines the deviation of the particle in the macromolecule. It specifies the protein structure flexibility and rigidity. The residues with higher peaks belong to loop areas or N- and C-terminal zones, as typically N and C fluctuate the most, recorded by MD trajectories (Figure 8). The stability of ligand binding to the protein is shown by low RMSF values of binding site residues. The percentages of Helix and Strand in vemurafenib–B-Raf kinase were determined to be 30.30% and 15.44%, respectively, and the overall secondary structure elements (SSE) were found to be 45.76%. In the case of vieloplain-F–B-Raf kinase, the percentages of Helix and Strand were 30.19% and 15.24%, respectively, and the total SSEs were 45.44 %. Protein SSEs are more rigid than the unstructured part of the protein, showing slight fluctuations in Figure 9.

#### 2.11.3. Protein–Ligand Interaction

The interaction of the target protein with the ligand was monitored during the simulation. These interactions were categorized into four types: (i) hydrogen bonds, (ii) hydrophobic, (iii) ionic, and (iv) water bridges.

**Figure 10:**
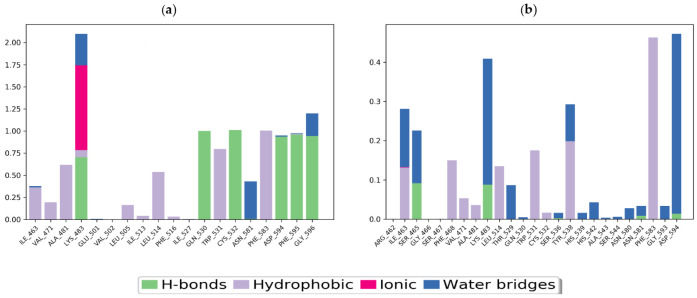
Protein–ligand contact histogram: (**a**) vemurafenib–B-Raf kinase, (**b**) vieloplain-F–B-Raf kinase.

This study found that the most significant ligand–protein interactions were hydrogen bonds, water bridges, and hydrophobic interactions. The vemurafenib–B-Raf Kinase complex showed the most important hydrophobic interactions with ALA_481, LEU_514, TRP_531, and PHE_583, whereas GLN_530, CYS_532, ASP_594, PHE_595, and GLY_596 were chief in terms of H-bonds (Figure 10A). In vieloplain-F–B-Raf kinase, the hydrophobic contacts PHE_583, TYR_538, TRP_531, LEU_514, ARG_462, and PHE_468 were the most vital, while SER_465 and LYS_483 were dynamic interactions for H-bonds (Figure 10b).

#### 2.11.4. RoG Analysis

**Figure 11:**
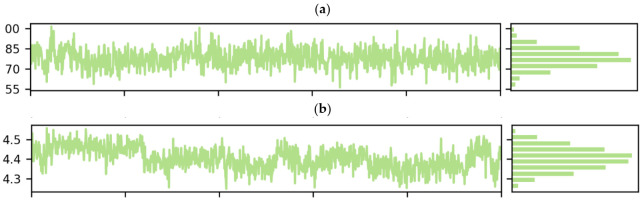
The radius of gyration was calculated for (**a**) vemurafenib–B-Raf kinase, (**b**) vieloplain-F–B-Raf kinase.

The folding and compactness of the protein are often arbitrated with the assistance of RoG. It is a crucial method of showing the influence of ligand on the three-dimensional conformational structural changes after the interaction with ligand. RoG with high valve depicts the molecule loose packing and folding nature of the protein after the interaction with the ligands. Both the complexes were in their native structures as there was not much variation observed throughout the 100 ns in the RoG graph (Figure 11). However, there were a few minor variations in RoG due to conformational changes in the secondary structure of protein during the MDS process. The RoG graph of the complexes shows that the ligand remains tightly bound to the protein’s active site.

### 2.12. MM-GBSA Calculations

**Figure 12:**
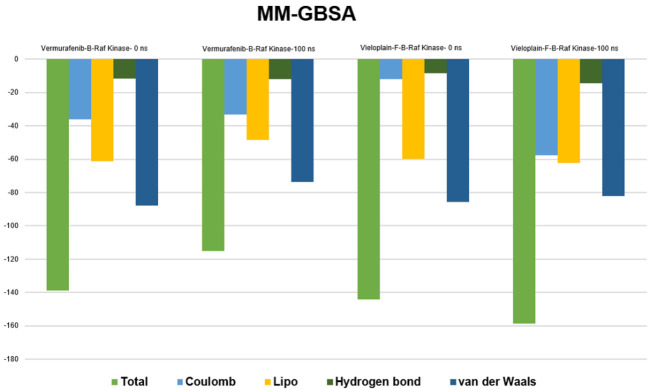
MM-GBSA calculated before and after the simulation.

The average binding free energy (ΔG) of the vemurafenib- B-Raf kinase and vieloplain-F-B-Raf Kinase was calculated at 0 and 100 ns by the MM-GBSA approach (Figure 12). The average values of ΔG for ligands vemurafenib were −138.8836 kcal/mol (0 ns) and −115.1949 kcal/mol (100 ns). The average dG for vieloplain-F were −144.2660 kcal/mol (0 ns) and −158.7822 kcal/mol. It was found that both compounds showed minimum VDW and hydrogen bonding energies that meant all these drugs are maximum potential to bind with active site residues.

## 3. Discussion

This work examines the utility of selecting vieloplain F as a possible biomolecule therapeutically active against melanoma by using publicly available techniques to investigate its bioactivity, ADMET, drug-likeness, molecular docking, and simulations. In silico interpretations of pharmacological spectra illustrated a new road to the most promising directions while also assisting in the initial phases of research by filtering out the biomolecules with a potentially low pharmacological profile [30]. According to the PASS prediction, vieloplain F could be considered an anti-neoplastic and anti-leukemic agent as sesquiterpene-type guaiane dimers are already reported for their attractive potencies against cancer [12,15] and reversal of resistance towards cancer [16]. Furthermore, the compound vieloplain F was also predicted as a testosterone 17-beta-dehydrogenase (NADP+) inhibitor, which means that vieloplain f could also be investigated for its participation against androgen and estrogen metabolism. The compounds also predicted high scores for NADP+ and evidenced by our studies that this compound has good effects against melanoma and it can be considered for its studies against different kind of skin diseases.

ADMET is crucial for every biomolecule before its biotransformation into a drug [21]. According to the ADMET profile of vieloplain F, the absorption and distribution of the compound were moderate. The compound is highly soluble in GIT and has no solubility for BBB, showing that this compound cannot create any adverse effects related to CNS. The inactive compounds on the CNS should not intersect to avoid adverse effects on the CNS [22]. The compound vieloplain F has a high ratio of GIT absorption as compared to control vemurafenib.

Furthermore, vieloplain F revealed that it is not a P-gp (P-glycoprotein) substrate; therefore, vieloplain F is not susceptible to the efflux mechanism of P-gp, which many cancer cell lines utilize as a drug resistance mechanism. CYP enzymes play a crucial role in drug excretion, and these isoforms are metabolizing almost 75% of market available drugs. Inhibition of any of these isoforms results in causing some significant pharmacokinetics-based drug-drug interactions [12,24]. Vieloplain F did not inhibit any of the CYP enzymes, but the control vemurafenib inhibited 3 CYP enzymes which meant that it can create drug-drug interactions for those CYP enzyme-targeted drugs. One of the significant drawbacks of vemurafenib was its causing hepatotoxicity [31] and cardiotoxicity [32]. The compound vieloplain F did not show any hepatotoxicity and is revealed to be cardioprotective. Blocking the hERG K^+^ channel can cause QT prolongation and potentially fatal arrhythmia [33]. As a result, vieloplain F was projected to be a non-inhibitor of hERG with no cardiac adverse effects. Finally, the toxicity profile acquired from pkCSM was adequate.

Acute toxicity was defined as the harmful effects of a single exposure to a drug over a short period [34]. In general, mice and rats were used to measure acute toxicity. The compound vieloplain F was predicted as non-toxic and categorized in class 4 with harmful indications if swallowed, suggesting the possible safe application. Furthermore, the compound vieloplain F did not exhibit any environmental toxicity violations.

CYP can act on aromatic or double bonds, probably leading to epoxy metabolites. Identifying a molecule’s epoxidation site will help guide improvement to avoid epoxidation for safer drugs [35]. Besides, reactive metabolites can cause idiosyncratic adverse drug reactions and drug-induced liver injury [36]. Predicting epoxy and reactive metabolites can thus help predict potential adverse effects. The present study shows that vieloplain F has been doubtful to result in epoxides and reactive metabolites compared to control vemurafenib.

An endocrine disruptor is a substance that operates on nuclear receptors to disrupt the endocrine or hormonal systems [37]. Compounds are docked into eighteen integrated and well-validated crystal structures of fourteen different human nuclear receptors using Endocrine Disruptome’s free web tool. As a result, achieving endocrine disruption potency for multiple targets at once is quite convenient. This eventually leads to rapid, well-informed judgments for in vitro and in vivo testing [38]. Vieloplain F was predicted to be non-endocrine disruptive, ensuring that it may be used safely. Among both compounds, including vieloplain F and vemurafenib, the highest profile for safety was achieved by vieloplain F.

To discover new anticancer drugs, sophisticated laboratory models are needed to predict their clinical behavior. This is mainly done from cell lines originating from human tumors [39]. In silico prediction will make the way more comfortable by offering useful predictions for making an informed decision before going into the In vitro experiments. The inhibitory effect of *Xylopia vielana* on cancer cell lines has been previously reported [13,15,16].

Vieloplain F has been studied as an anticancer agent in our recent study and has demonstrated potent activity against prostate cancer cell lines with an IC_50_ value of 9.5 µM [15]. However, particular attention to the deep mechanism has not yet been drawn. The present study demonstrated a specific targeted approach that can be useful in further anticancer drug development of B-Raf kinase.

B-Raf is a member of the Raf-kinase family of growth signal transduction protein kinases. This protein plays a role in regulating the MAP kinase/ERKs signaling pathway, affecting cell division, differentiation, and secretion. B-Raf kinase inhibitors are also considered one of the main treatments for malignant melanomas. Currently, only one B-Raf kinase inhibitor, vemurafenib, is approved by the FDA and is used in late-stage melanoma [39], but the main drawback of this drug is that patients start showing symptoms after receiving this therapy resistance towards this drug [8]. Therefore, research is underway to develop novel B-Raf kinase inhibitor compounds with maximum potency and minimum side effects. There is still scope to develop B-Raf kinase inhibitors with improved therapeutic efficacy and reduced side effects. As evident from our study, vieloplain F showed encouraging results in terms of B-Raf kinase inhibition compared to vemurafenib in the sense of molecular docking and simulation studies. Therefore, in these ways, special attention should be placed on investigating the therapeutic importance of this process.

Thus, the present study revealed the potential significant applications of vieloplain F and could be helpful against various diseases. The study relied on computational tools that reported pharmacological properties and bioactivities predictions. Besides, in this study, we reported the potentials of vieloplain F with comparison studies on vemurafenib, and in previous studies, the in vitro and in vivo potential of vieloplain F was displayed. Moreover, clinical studies are necessary to confirm the findings of the present work. Nonetheless, the results of this work will provide future guidance for the design and development of new lead compounds against Melanoma.

## 4. Materials and Methods

### 4.1. Isolation of Guaiane Dimer Vieloplain F

Isolation procedures for guaiane dimer, vieloplain F, and its structure elucidations based on spectroscopic methods (1D, 2DNMR, X-ray, and ESI-MS) along with a comparison of the relevant literature data reported for guaiane dimer, vieloplain F, were described in detail in our recent paper [15]. 

### 4.2. Prediction of Activity Spectra for Substances (PASS) 

Prediction of activity spectra for substances (PASS) gives information about the bioactive compounds’ plausible pharmacological activities. The free internet platform Pass online (http://www.pharmaexpert.ru/passonline, accessed on 10 August 2021) was used to make the PASS prediction. Only actions with Pa > Pi were considered feasible for a given compound. Pa > 0.7 indicated a high probability of experimental pharmacological effect, while Pa 0.5 to 0.7 indicated a moderate probability of experimental pharmacological action. If Pa was less than 0.5, the chances of pharmacological activity were negligible [40].

### 4.3. ADMET Analysis

ADMET (absorption, distribution, metabolism, excretion, and toxicity) are the essential measurement tools for any compound before being elected as a drug candidate. The online web tool swiss ADME (http://www.swissadme.ch/index.php, accessed on 10 August 2021) was used to obtain ADME properties of the vieloplain F [24], and the pharmacokinetic scores were predicted using the online web application pkCSM (http://biosig.unimelb.edu.au/pkcsm/prediction, accessed on 10 August 2021).

### 4.4. Prediction of Acute Rat Toxicity and Environmental Toxicity

The publicly accessible structure–activity relationship (GUSAR) software (http://www.way2drug.com/gusar/acutoxpredict.html, accessed on 10 August 2021) was used to predict median lethal dosage (LD_50_) values for rats with oral administration [41]. The quantitative predictions of ecotoxicity were also assessed by GUSAR software (http://www.way2drug.com/gusar/environmental.html, accessed on 10 August 2021).

### 4.5. Prediction of Drug-Likeness

Swiss ADME (http://www.swissadme.ch, accessed on 10 August 2021) and Molinspiration Chemoinformatics tools (https://www.molinspiration.com/cgi-bin/properties, accessed on 10 August 2021) were used to predict drug-likeness. Lipinski’s rule of five was considered a standard for accessing the drug-likeness [27].

### 4.6. Bioactivity Score Prediction 

The bioactivity profile of a selected compound can be portrayed by the scoring system of G protein-coupled receptor (GPCR) ligand, ion channel modulator, nuclear receptor legend, a kinase inhibitor, protease inhibitor, and an enzyme inhibitor. These properties were determined by Molinspiration Chemoinformatics tools (https://www.molinspiration.com/cgi-bin/properties, accessed on 10 August 2021). According to studies by Roy, if the value was equal to or greater than 0.00 (≥0), the compound was more active, while if the values were between −0.50 and 0.00, it was moderately active; nevertheless, if the values were less than −0.50 (<−5.0), it was thought to be inactive [42].

### 4.7. Prediction of Cardiac Toxicity

The blockage of the hERG K^+^ channels has been linked to fatal cardiac arrhythmias. The pred-hERG 4.2 (http://predherg.labmol.com.br, accessed on 10 August 2021) web server, a web tool for early detection of putative hERG blockers and non-blockers, was used to predict cardiac toxicity [38].

### 4.8. Prediction of Epoxidation and Reactivity to Biological Macromolecules

Epoxides are metabolites produced by an enzyme cytochrome P450 operating aromatic or double bonds. Drug-metabolizing enzymes can bioactivate the drug into reactive metabolites, creating adducts when they bind to specific targets in DNA or proteins. The freely available web Xenosite (https://swami.wustl.edu/xenosite/submit, accessed on 10 August 2021) was used to predict epoxidation and reactivity to biological macromolecules [43].

### 4.9. Prediction of Endocrine Disruption Potential

Endocrine Disruptome is an unrestricted prediction tool for determining the potential for endocrine disruption via nuclear receptor binding. Fourteen human nuclear receptors and their eighteen validated structures that regulate reproduction, behavior, development, metabolism, and the immune system were utilized for molecular docking with the compounds in the freely accessible web platform Endocrine Disruptome (http://endocrinedisruptome.ki.si, accessed on 10 August 2021) [20]. 

### 4.10. Prediction of Cell Line Cytotoxicity

CLC-Pred (Cell Line Cytotoxicity Predictor) is a web-based program that predicts the cytotoxicity of chemical compounds in non-transformed and cancer cell lines depending on their structural formula. Prediction of cell line cytotoxicity of vieloplain F was made through CLC-Pred (http://www.way2drug.com/Cell-line/, accessed on 10 August 2021). The predicted output activity was represented in the probable activity (Pa) and probable inactivity (Pi) score. The scoring system was categorized into three portions according to activity. Pa > 0.5 was considered as the highest activity, Pa > 0.3 was considered as moderate activity, and Pa < 0.3 was considered as the lowest activity. [44].

### 4.11. Preparation, Analysis, Retrieval, and Visualization of Protein and Ligand Structures

ChemBioDraw (PerkinElmer Informatics, Waltham, MA, USA, v13.0) [45] was used to draw the compounds into .mol format. The control drug vemurafenib was downloaded from PubChem in .sdf file. The three-dimensional (3D) structures of B-Raf Kinase was accessed from Protein Data Bank (PDB) (www.rcsb.org, accessed on 10 August 2021) with PDBIDs 3OG7. The selected protein structure was minimized using Chiron portal (https://dokhlab.med.psu.edu/chiron/processManager.php, accessed on 10 August 2021) and visualized through UCSF Chimera 1.10.1 tool [46]. The Ramachandran plots of B-Raf kinase were accessed from the Discovery Studio 4.1 Client tool. The protein architecture and statistical percentage values of receptor proteins helices, beta-sheets, coils, and turn were predicted from online server VADAR 1.8 (http://vadar.wishartlab.com/, accessed on 10 August 2021) [47].

### 4.12. Molecular Docking 

Docking studies of vieloplain F and vemurafenib were performed against B-Raf Kinase. To prepare the B-Raf Kinase structure, the unnecessary ligands and water molecules were removed to enhance docking results’ efficacy. The ligands were sketched in the ACD/ChemSketch 2.1.2 tool (Advanced Chemistry Development, Toronto, Canada) and further minimized by UCSF Chimera 1.10.1. A docking experiment was used on all synthesized compounds against B-Raf Kinase using the PyRx docking tool 1.7 (https://pyrx.sourceforge.io/, accessed on 10 August 2021) [48]. To perform the docking experiment, grid box parametric dimension values were adjusted as X = −1.3845, Y = −12.9405, and Z = −18.9916, respectively. The default exhaustiveness = 8 value was used to obtain the finest binding conformational pose of protein-ligand docked complexes. All compounds were docked separately against the crystal structure of B-Raf kinase. The docked complexes were evaluated on lowest binding energy (kcal/mol) values, hydrogen and hydrophobic bond interaction pattern analysis using Discovery Studio (4.1) (Dassault Systemes BIOVIA, San Diego, CA, USA), and UCSF Chimera 1.10.1. The three-dimensional (3D) graphical depictions of all the docked complexes were accomplished by Discovery Studio (2.1.0) (Dassault Systemes BIOVIA, San Diego, CA, USA) and the UCSF Chimera 1.10.1 tool. 

### 4.13. Molecular Dynamics Simulations

The MDS analysis was performed to find the interaction of ligand–protein stability. MD simulation studies also analyze the structure of the macromolecules transition to the functional significance of the complex. Simulation typically records atom movement concerning the time based on Newton’s standard motion equation to predict the binding of the ligand in the biological environment. The MD simulation of the selected complex was undergone for 100 ns using Desmond v:3.6 New York, NY, USA module from Schrodinger suite [12]. The interaction of the complex obtained from MD was the initial structure of respective MD simulations followed by an established protocol where protein atoms were 10 Å away from the box. The ligand-receptor complex minimization and optimization were done through Wizard of Maestro (Schrödinger, New York, NY, USA). The systems were set up by applying the System Builder tool, a solvent standard TIP3P with an orthorhombic box was selected. The OPLS 2005 was used for simulation analysis. The Physiologic conditions of the model were minimized by adding 0.15 M NaC [49]. The models were rested before the start of the simulation. Lastly, simulations were run at 300 K temperature at 1 atm pressure, with an NPT ensemble was applied for all MDSs.

Moreover, the MDS trajectories were recorded after every 100 ps interval. The root means square deviation (RMSD) of both the protein and ligand were recorded to find the stabilities of simulation. The RMSF and RoG values were also calculated. MDSs were repeated thrice for each complex using the same parameters.

### 4.14. MMGBSA Calculations

The ligand-receptor complexes were minimized using the prime tool in maestro. After minimization, the molecular mechanics generalized born surface area (MMGBSA) was used to evaluate the binding free energies (∆G) of complexes at both before (0 ns) and after (100 ns) simulation. All over the computation of binding free energies, an OPLS_2005 force field was utilized [50].

## 5. Conclusions 

Natural products of herbal origin are well known to exhibit diverse biological activities compared to synthetic products. Here, in this study, one potent guaiane dimer, vieloplain F, was isolated from *Xylopia vielana* species and tested against B-Raf kinase to find a potent drug molecule against melanoma. The compound showed potent inhibitory activity against the B-Raf kinase protein receptor. The preliminary computational studies such as ADMET, bioactivities, and molecular docking studies proved that this guaiane dimer has a high binding affinity towards the targeted B-Raf kinase protein receptor. In addition, it revealed that our isolated natural compound is safer than the FDA-approved drug vemurafenib in cardiac and hepatotoxicity profile and has high binding energies towards targeted protein then vemurafenib. Overall, the present study acts as evidence to prove that this guaiane dimer isolated from the *Xylopia vielana* has the capacity to inhibit the B-Raf kinase protein receptor, which also opens the road for all the guaiane dimers that all these compounds should be screened for B-Raf kinase protein. The in silico studies can provide a platform for a potential compound against any specific disease but still, before any biomolecule needs to be selected, further studies must pass through deep in vivo and in vitro studies to confirm their results. The isolation methods, the quantity of the pure compound, and the complex structures of the natural products create a big question for future researchers to resolve this problem and do the wetlab assays independently.

In this study, the complete pharmacological profile encompassing PASS, bioactivity scores, ADMET, molecular docking, and molecular simulations will act as a foundation for other guaiane dimers to be investigated in the future for different types of cancers.

## Figures and Tables

**Table 1:** Best predicted bioactivities of compounds by PASS online.

Biological Activities	Pa	Pi
Anti-neoplastic	0.862	0.006
Anti-leukemic	0.592	0.009
Testosterone 17beta-dehydrogenase (NADP+) inhibitor	0.589	0.094

**Table 2:** Predicted physicochemical parameters and lipophilicity properties of vieloplain F and vemurafenib.

Properties	Parameters	Vieloplain F	Vemurafenib
Physicochemical Properties	MW ^a^ (g/mol)	446.62	489.92
	Rotatable bonds	0	7
	HBA ^b^	3	6
	HBD ^c^	0	2
	Fraction Csp3	0.63	0.13
	TPSA ^d^	51.21	100.30
Lipophilicity Log *P_o/w_*	iLOGP	3.93	3.04
	XLOGP3	4.02	4.97
	MLOGP	6.35	3.41
	Consensus	5.07	4.89

^a^ Molecular weight, ^b^ H-bond acceptor, ^c^ H-bond donor, ^d^ Topological polar surface area.

**Table 3:** Predicted pharmacokinetics parameters of vieloplain F and vemurafenib.

Properties	Parameters	Vieloplain F	Vemurafenib
Absorption	Water solubility	−5.971	−4.656
	GI ^a^	100	98.45
	Log *K*_p_ (skin permeation) cm/s	−6.17	−5.76
Distribution	BBB ^b^	−0.37	−1.686
	CNS permeation (Log PS)	−1.027	−2.976
	V_D_^c^ (human)	−0.013	−0.461
Metabolism CYP2D6	CYP1A2 inhibitor	No	No
	CYP2C9 inhibitor	No	Yes
	CYP2C19 inhibitor	No	Yes
	CYP3A4 inhibitor	No	Yes
	CYP2D6 inhibitor	No	No
Excretion	Total Clearance (log mL/min/kg)	0.053	0.136
	Renal OCT2 substrate	No	No

^a^ Gastrointestinal, ^b^ Blood-brain barrier, ^c^ Volume of distribution.

**Table 4:** Predicted toxicity profile of vieloplain F and vemurafenib.

Parameters	Vieloplain F	Vemurafenib
Ames Toxicity	No	No
Max. Tolerated Dose (human) (log mg/kg/day)	0.013	0.601
hERG I Inhibitor	No	No
hERG II Inhibitor	Yes	Yes
Oral Toxicity (LD50) (mg/kg)	1640	2316
Oral Toxicity classification *	IV	V
Hepatotoxicity	No	Yes
Skin Sensitization	No	No
Bioaccumulation Factor Log_10_ (BCF)	2.489	0.674
*Daphnia magna* LC_50_ − Log_10_ (mol/L)	7.127	6.969
Fathead Minnow LC_50_·Log10 (mmol/L)	−4.972	−4.154
*Tetrahymena pyriformis* IGC_50_ − Log_10_ (mol/L)	1.998	2.203

* Class I: fatal if swallowed (LD_50_ ≤ 5); class II: fatal if swallowed (5 < LD_50_ ≤ 50); class III: toxic if swallowed (50 < LD_50_ ≤ 300); class IV: harmful if swallowed (300 < LD_50_ ≤ 2000); class V: may be harmful if swallowed (2000 < LD_50_ ≤ 5000); and class VI: non-toxic (LD_50_ > 5000).

**Table 5:** Bioactivity score of the compounds vieloplain F and vemurafenib according to the Molinspiration software.

Compounds	GPCR Ligand	Ion Channel Modulator	Kinase Inhibitor	Nuclear Receptor Ligand	Protease Inhibitor	Enzyme Inhibitor
Vemurafenib	0.45	0.25	0.64	0.02	0.12	0.34
Vieloplain F	−0.12	−0.18	−0.54	0.17	−0.04	0.06

**Table 6:** In silico prediction of cell line cytotoxicity by CLC-pred.

Compound	Cell Line	Cell Line Full Name	Tissue	Tumor Type	Pa	Pi
Vemurafenib	SK-MEL-28	Melanoma	Skin	Melanoma	0.618	0.010
	A2058	Melanoma	Skin	Melanoma	0.581	0.004
	M14	Melanoma	Skin	Melanoma	0.546	0.012
	PA-1	Ovarian carcinoma	Ovarium	Carcinoma	0.520	0.005
Vieloplain F	SK-MEL-2	Melanoma	Skin	Melanoma	0.722	0.006
	K562	Erythroleukemia	Hematopoietic and lymphoid tissue	Leukemia	0.598	0.015

**Table 7:** Chemical structures, amino acid residues, and docked results of the vieloplain F and vemurafenib with B-Raf kinase (PDB: 3OG7).

Compound Name	Chemical Structure	Binding Energy (kcal/mol)	Amino Acid Residues Involved in the Bonding
Vieloplain F	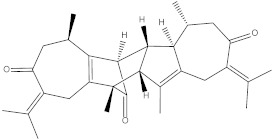	−11.8	Ser164, Asp165, Asp125, Lys127, Trp150, Ser147, Ile148, Met151, Val155.
Vemurafenib	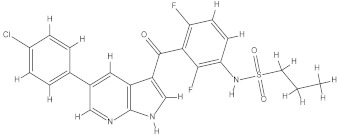	−10.2	Asp143, Lys127, Asn129, Ser88, Phe132, Ser87, Cys84, Trp83, Ala33, Ile15, Ile79, Lys35, Phe20.

## Data Availability

Not applicable.

## Supplementary Materials

The following are available online: Figure S1: Bioavailability radar chart for vemurafenib, Figure S2: Ramachandran graph of target protein, Table S1: Drug-likeness profile of Vieloplain F and Vemurafenib.
