# Supplementary material for: A Comprehensive In Silico Exploration of Pharmacological Properties, Bioactivities, Molecular Docking, and Anticancer Potential of Vieloplain F from Xylopia vielana Targeting B-Raf Kinase
Source: Molecules. 2022 Jan 28;27(3):917. doi: 10.3390/molecules27030917 (PMC8839023; doi:10.3390/molecules27030917)
Supplement: Supplementary file 1 [file molecules-27-00917-s001.zip › molecules-1550514-supplementary.pdf]

molecules

Supplementary Materials

A Comprehensive In Silico Exploration of Pharmacological Properties, Bioactivities, Molecular Docking, and Anticancer Potential of Vieloplain F from *Xylopia vielana* Targeting B-Raf Kinase

Syed Shams ul Hassan [1,2], Syed Qamar Abbas [3], Fawad Ali [4], Muhammad Ishaq [1,2], Iqra Bano [5], Mubashir Hassan [6,7], Hui-Zi Jin [1,2,*] and Simona G. Bungau [8]

[1] Shanghai Key Laboratory for Molecular Engineering of Chiral Drugs, School of Pharmacy, Shanghai Jiao Tong University, Shanghai 200240, China; Shams1327@yahoo.com (S.S.u.H.); mishaqjnj@yahoo.com (M.I.).
[2] Department of Natural Product Chemistry, School of Pharmacy, Shanghai Jiao Tong University, Shanghai 200240, China.
[3] Department of Pharmacy, Sarhad University of Science and Technology, Peshawar 25000, Pakistan; Qamar0613@yahoo.com.
[4] Department of Pharmacy, Kohat University of Science and Technology, Kohat 26000, Pakistan; fawadalee@gmail.com.
[5] SBBUVAS, Faculty of Bio-sciences Sakrand,67210 Sindh, Pakistan; iqrashafi05@yahoo.com.
[6] Institute of Molecular Biology and Biotechnology, The University of Lahore, Nisbet road, Lahore 54000, Pakistan; Mubashirhassan_gcul@yahoo.com.
[7] Battelle Center for Mathematical Medicine, The Research Institute at Nationwide Children's Hospital, Columbus, OH 43205, USA.
[8] Department of Pharmacy, Faculty of Medicine and Pharmacy, University of Oradea, 410028 Oradea, Romania; simonabungau@gmail.com.
* Correspondence: Kimhz@sjtu.edu.cn; Tel.: (0086-130-65711080)

Figure S1 Bioavailability radar chart for vemurafenib.
Figure S2 Ramachandran graph of target protein
Table. S1 Drug-likeness profile of Vieloplain F and Vemurafenib.

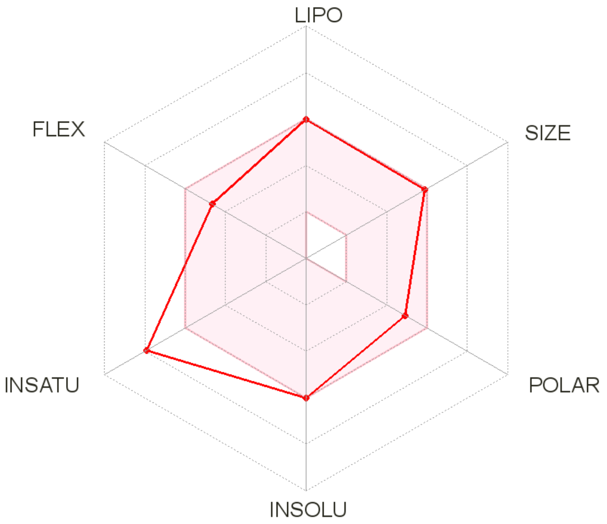

Figure S1. Bioavailability radar chart for vemurafenib.

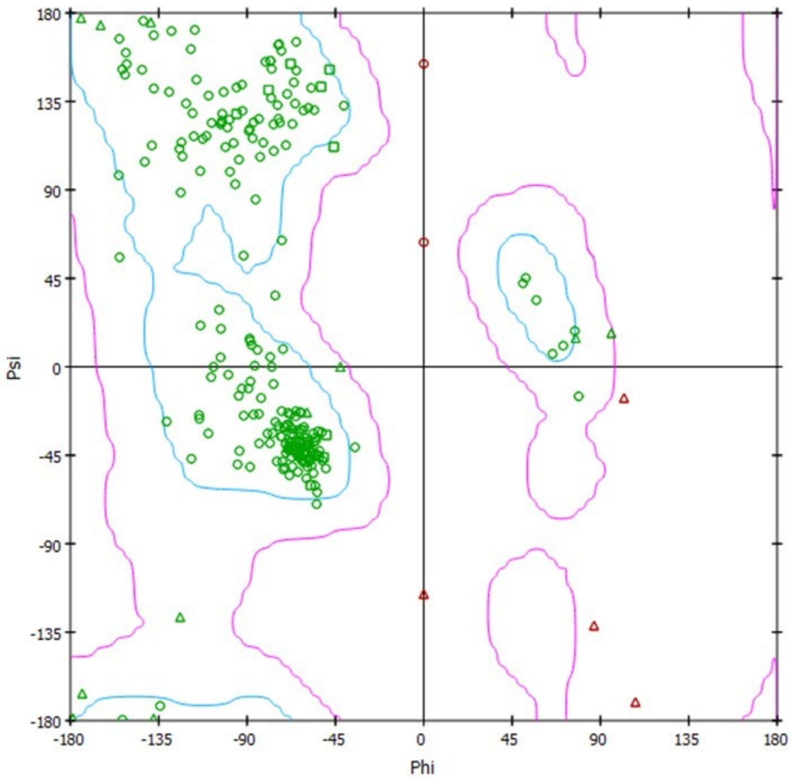

Figure S2. Ramachandran graph of target protein.

Table S1. Drug-likeness profile of Vieloplain F and Vemurafenib.

Compounds	Drug Likeness		Medicinal Chemistry	
	Lipinski violations	VEBER violations	PAINS alerts	Synthetic accessibility
Vemurafenib	0	0	0	3.38
Vieloplain F	1	0	0	6.46
